# Lectures on the Diagnosis and Treatment of Diseases of the Heart

**Published:** 1841-12-25

**Authors:** W. W. Gerhard


### MEDICAL EXAMINER.

DEVOTED TO MEDICINE, SURGERY, AND THE COLLATERAL SCIENCES.

No. 52.] PHILADELPHIA, SATURDAY, DECEMBER 25, 1841. [Vol. IV.

### LECTURES ON THE DIAGNOSIS AND TREATMENT OF DISEASES OF THE HEART.

BY W. W. GERHARD, M. D.

### LECTURE XXII.

#### DILATATION.

This is enlargement of the cavities of the heart. One variety of it is, as we have already seen, connected with thickening of the parietes of the heart, and is, therefore, properly considered as belonging to hypertrophy rather than to dilatation; but it also occurs as a distinct lesion, and then the parietes of the heart are in general not only free from thickening, but are rather thinner than natural. It is in itself by no means a formidable disease, but as it often is connected with different organic lesions, or with embarrassing nervous disorder, it may indirectly prove to be a source of great annoyance and even danger to the patient.

The causes of dilatation are nearly the same as those of hypertrophy, but, in the latter disease, the general state of the individual is more disposed to active nutrition of a semi-inflammatory kind, while in dilatation the force of resistance of the heart is in a great degree lost, and the organ becomes thinner and weaker. Hence it is more frequent in anemic individuals, especially in chlorotic girls, than in any other class of persons, just as hypertrophy is most frequent in males. The tissue of the heart is paler and more flaccid than in thickening, and there is very rarely that complication of endo or pericarditis which renders the membrane of a hypertrophied heart so often opaque and thickened. The degree of dilatation is very various; it never, in the simpler variety, reaches nearly to the degree observed in the cases complicated with hypertrophy, for the plain reason, that the powers of the muscular structure of the heart would yield to the continued stretching unless it were increased in thickness: hence there is a natural law of the economy that a dilated hollow organ tends to hypertrophy. Dilatation then often ceases to be simple, from the natural operation of this law. The organic lesions most frequently conjoined with dilatation, are the disorders of the valves, especially the auriculo-ventricular. These are more frequently extreme patescence than thickening and contraction, and in some cases of dilatation the symptoms begin so suddenly, and with so much force from the first, that it would seem that one or more of the chordiæ tendineæ had given way, producing a sudden inability of using the valves.

*Symptoms.*—The physical signs are less marked than those of hypertrophy. The percussion is rarely dull to the same extent, as it depends merely upon the blood contained in the heart, and not on the addition of solid muscular structure: it must, however, necessarily be more dull than usual, and the dulness will extend over a greater space. There is no prominence, for the heart has not force enough to act upon the ribs and oblige them to recede. The chief signs, however, of dilatation are the changes in the sounds of the heart. The thinness of the parietes increases the sharpness and loudness of the first sound, much as the thickening of hypertrophy renders it dull and less distinct; it therefore approaches, to a certain extent, the sharpness of the second sound. The latter is also increased, and becomes much more clacking than it is in the healthy heart. The rapidity of the heart's action is in most cases increased, sometimes to a very great degree, and slight causes produce palpitation.

Pain is not unfrequently complained of by the patient; sometimes it is sharp, but in general it is like the pain in most cases of organic disease of the heart, that is, dull and indistinct. When the pain is most severe, the case is generally complicated with functional disease of the heart. The disturbances of the capillary circulation of various organs, which are so apt to occur in hypertrophy, rarely take place in simple dilatation, so that the vascular symptoms of this disease are less evident.

*General Symptoms.*—As dilatation occurs chiefly in individuals of a nervous temperament, often more or less anemic, many disturbances are apt to occur in the nervous condition of the patient; hence neuralgia, in its various forms, and hysteria are frequent complications. There is no other symptom properly dependant upon the disease, but a large number of functional disorders may either precede or accompany it, and modify the action of the heart. Hence complications of this kind are purely or nearly accidental. The most important is the general feebleness of the patient; as long as this continues, the action of the heart is rarely restored to its normal state, for the functional disturbance keeps up, as it were, the symptoms of dilatation.

*Diagnosis and Prognosis.*—The diagnosis of dilatatien, and the organic diseases of the heart, is not very difficult, but it is often extremely embarrassing to decide between it and nervous disorder, that is, to ascertain how much of the symptoms is owing to each of these causes. The distinction depends on the signs of dulness found in dilatation, and the permanent loudness of the sounds of the heart; still it will often be exceedingly difficult to decide whether a heart which is the subject of great functional disorder is dilated or not. A hasty opinion must not be formed under such circumstances; but, by attention to the progress of the disorder, its true character will be developed. In this, as in other diseases of the heart, the functional affections are more or less temporary, the organic alterations are much more permanent, and, although they may subside for a time, they do not entirely disappear, unless a real recovery should occur. The prognosis of the disorder offers little of interest; it is scarcely fatal of itself, but, as connected with other disorders, it may undoubtedly exercise a certain agency in favouring and hastening their progress. A patient scarcely ever dies of simple dilatation.

*Treatment.*—In dilatation, as well as in hypertrophy, the treatment is not active, so far as our means of acting directly upon the heart extend. Our object is to tranquillize the action of the heart, and to support the strength of the patient, if, as often happens, it be much enfeebled. The pain which sometimes attends dilatation may be relieved by small blisters over the region of the heart, and to the spine, in those cases in which the action of the heart is in a great degree kept up by spinal irritation. Blistering, or other revulsives to the spine, may cure the patient of the palpitation, and the dilatation will then present few symptoms of importance. The action of the heart in dilatation is not powerful enough to require the use of large doses of digitalis; if this remedy be administered, it should be given only for a short period, and in very minute doses, conjoined with aq. camphoræ, or lac assafœtidæ. Hoffman's anodyne answers very well for the same object, as a temporary tranquillizing remedy. The use of these various remedies is readily learned when it is remembered that the power of the heart should not be diminished, but that its irregular action merely should be checked. For the same purpose a rigid adherence to hygienic rules becomes necessary, and they should be nearly the same as those required for the treatment of hypertrophy. But as the nervous stimulants are more to be dreaded in dilatation than the more permanent excitants of the heart, the patient should carefully abstain from tobacco, tea, coffee, and avoid the mental and physieal excesses which disturb the regular cardiae action. Those which act through the passions, or debilitate the nervous power by excessive fatigue from indulgence in sensual gratification, or undue culture of some of the faculties, are the most injurious.

If anemia complicates dilatation of the heart, a long continued use of tonics, especially the chalybeate, constitutes the best mode of treatment. The prescription which I prefer as a general rule is Valet's proto-carbonate of iron, in doses of two or three grains, with half a grain of rhubarb and a grain of ginger; of these pills the patient may take from three to four daily. The infusion of the Prunus Virginiana is an adjuvant to the chalybeate remedies of great value, and often succeeds in quieting the action of the heart. Cold bathing, sea bathing, and other tonics of this nature, are all necessary from time to time, and may be prescribed with advantage, as they tend to strengthen the general muscular system, and to increase the nutrition, without exciting the irritability of the heart.

---

This lecture brings to a close the series on diseases of the chest by Dr. Gerhard, published in this journal."

